# Rho GTPases: Novel Players in the Regulation of the DNA Damage Response?

**DOI:** 10.3390/biom5042417

**Published:** 2015-09-30

**Authors:** Gerhard Fritz, Christian Henninger

**Affiliations:** Institute of Toxicology, Heinrich Heine University Düsseldorf, Moorenstrasse 5, 40225 Düsseldorf, Germany; E-Mail: henninger@uni-duesseldorf.de

**Keywords:** DNA damage response, Rho GTPases, genotoxic stress, HMG-CoA reductase inhibitors (statins), anticancer drugs, normal tissue damage, chemical carcinogenesis

## Abstract

The Ras-related C3 botulinum toxin substrate 1 (Rac1) belongs to the family of Ras-homologous small GTPases. It is well characterized as a membrane-bound signal transducing molecule that is involved in the regulation of cell motility and adhesion as well as cell cycle progression, mitosis, cell death and gene expression. Rac1 also adjusts cellular responses to genotoxic stress by regulating the activity of stress kinases, including c-Jun-N-terminal kinase/stress-activated protein kinase (JNK/SAPK) and p38 kinases as well as related transcription factors. Apart from being found on the inner side of the outer cell membrane and in the cytosol, Rac1 has also been detected inside the nucleus. Different lines of evidence indicate that genotoxin-induced DNA damage is able to activate nuclear Rac1. The exact mechanisms involved and the biological consequences, however, are unclear. The data available so far indicate that Rac1 might integrate DNA damage independent and DNA damage dependent cellular stress responses following genotoxin treatment, thereby coordinating mechanisms of the DNA damage response (DDR) that are related to DNA repair, survival and cell death.

## 1. Regulation and Biological Function of Rho GTPases

Rac1 belongs to the family of Ras-homologous (Rho) GTPases, which are member of the Ras superfamily of low molecular weight monomeric GTP-binding proteins [1,2,3]. Rho GTPases cycle between an active GTP-bound and an inactive GDP-bound state, thereby acting as molecular switches [4]. In their active form, they are localized at the inner side of the outer cell membrane. Upon activation they interact with various effector molecules, such as p21-activated protein kinases (PAK), IQGAP, PAR6 and p67phox [5,6,7,8] to initiate downstream responses. In general, Rho GTPases are activated upon binding of ligands to cellular growth factor and cytokine receptors as well as heterotrimeric G-protein coupled receptors [9,10]. GTP-binding is facilitated by *guanine nucleotide exchange factors* (GEFs), while intrinsic GTP hydrolysis is stimulated by *GTPase activating proteins* (GAPs). The interaction of Rho proteins with membranes requires a lipid moiety (geranylgeranylpyrophosphate or farnesylpyrophosphate residue), which is attached to the cysteine at their C-terminal CAAX box [11,12]. In the case that this prenyl group is masked by the *Rho guanine nucleotide dissociation inhibitor* (Rho GDI), cytosolic sequestration of Rho GTPases is favored [13,14]. Rho GDI also inhibits spontaneous GDP for GTP exchange and GTPase activity. Employing constitutively active (V12) and dominant negative (N17) Rho mutants as well as bacterial toxins, in particular toxins originating from different clostridium species (*i.e.*, *Clostridium botulinum*, *Clostridium difficile*, *Clostridium sordellii*) have provided detailed insight into Rho-regulated processes [15,16]. One major function attributed to Rho GTPases is the organization of the actin cytoskeleton [17], with formation of actin stress fibers by RhoA, actin-rich lamellipodia by Cdc42 and membrane ruffles by Rac1 being best characterized [18]. Actin cytoskeleton independent functions are also regulated in a Rho-dependent manner, including the Rac1-dependent activation of c-Jun-N-terminal kinases/stress activated protein kinases (JNK/SAPK) and p38 kinases [19,20], which are prototypical kinases activated by genotoxic stress [21] and regulate cell death [22,23]. Downstream of the aforementioned kinases, various transcription factors (including AP-1, ATF2 and nuclear factor kappa-B) get activated [21,24,25], which impact cell death mechanisms as well. Apart from regulating signaling pathways that get stimulated in response to endogenous and exogenous stress, Rho GTPases also play a key role in the regulation of mechanisms of malignant transformation, tumor growth and metastasis [26,27,28], including the regulation of E-cadherin-dependent cell-cell contacts [29], focal adhesions [30], G1/S phase progression [31] and apoptosis [32]. However, whether all of these functions of Rho GTPases predominantly depend on membrane-bound Rho proteins or whether nuclear Rho functions are involved as well is still enigmatic.

## 2. Rho GTPases in the Regulation of Genotoxic Stress Responses

Upon exposure to chemical or physical genotoxic noxae cells respond with a complex cellular program that either promotes or protects against cell death. These manifold stress responses can be classified into DNA damage dependent and DNA damage independent mechanisms. For instance, rapid stress responses to genotoxins such as UV-C light, alkylating agents or oxidants can originate from the outer cell membrane as a result of (i) the activation of growth factor receptors by inhibition of phosphatases [33,34,35,36,37] and (ii) non-receptor tyrosine kinases, in particular c-Abl, which is known to shuttle between cytoplasmic and nuclear compartments and regulates cell death following genotoxic insults [38,39,40]. It has been suggested that SAPK/JNK and c-Abl are part of a signaling network that is stimulated when DNA mismatch repair (MMR) proteins recognize distortions in the DNA, as induced for instance by cisplatin adducts [41]. Key events involved in the so-called immediate-early DNA damage independent stress response comprise the activation of SAPK/JNK and p38 kinase, belonging to the family of mitogen activated protein kinases (MAPK), and corresponding survival- or death-related transcription factors (e.g., AP1) [42]. Importantly, genotoxin-induced activation of SAPK/JNK is also affected by the DNA repair status of cells [41,43,44] and replicative stress causes rapid activation of SAPK/JNK, too [45]. This indicates that, apart from DNA damage independent mechanisms, DNA damage-related functions contribute to the activation of SAPK/JNK as well. It is feasible that specific signals arising from the outer membrane and the nucleus can integrate at specific molecules, such as SAPK/JNK, and that the nature of the agent and time after exposure are important factors determining the balance. Noteworthy, similar to the PI3-kinase-related kinases ATM/ATR, which are the key players in the regulation of the DNA damage response (DDR) activated by DNA double-strand breaks (DSBs) or replicative stress [46], SAPK/JNK is also able to phosphorylate histone H2AX at position S139 [47,48,49]. Phosphorylated H2AX (γH2AX) in turn provides the docking point for the association of numerous factors involved in DNA repair [50]. Thus, γH2AX appears to integrate signals originating from ATM/ATR and SAPK/JNK signaling pathways. Moreover, p38 kinase is known to form a complex with MAPK-activated protein kinase 2 (MK2) [51,52], which controls DNA damage-induced checkpoint mechanisms (*i.e.*, Chk1, Chk2) independent of ATM/ATR [53,54]. Taken together, Rac1 is required for the activation of stress kinases (*i.e.*, SAPK/JNK and p38), which play a pivotal role in the regulation of genotoxin-induced stress responses.

In order to further characterize the involvement of Rac1/Rho-regulated mechanisms in the regulation of cellular responses to genotoxins, Rho-inactivating clostridial toxins as well as pharmacological strategies have been employed. For instance, inactivation of Rac1 by use of clostridial toxins (*i.e.*, *Clostridium difficile* toxin B and lethal toxin (LT) from *Clostridium sordellii*) largely abrogated the activation of SAPK/JNK, as well as of NF-κB, following UV-C irradiation [55]. Regarding pharmacological approaches, data obtained from cardiovascular research have demonstrated that Rho GTPases, in particular Rac1, are the most relevant targets for the pleiotropic beneficial effects of HMG-CoA reductase inhibitors (statins) on the cardiovascular system [56,57,58]. By inhibiting the mevalonate pathway, statins deplete the pool of isoprene precursor molecules, which are not only required for cholesterol biosynthesis, but are also needed for C-terminal prenylation of Rho GTPases at their CAAX-box [57,59,60]. Therefore, apart from their lipid-lowering function, statins exhibit pleiotropic effects resulting from interference with Rho-signaling. Using statins as a pharmacological tool, it has been shown that these drugs effectively block the activation of SAPK/JNK by UV-C light, methylating agents and cisplatin [55,61,62,63].

## 3. Pharmacological and Genetic Targeting of Rac1-signaling Impacts Activation of DDR Mechanisms

**Figure 1:**
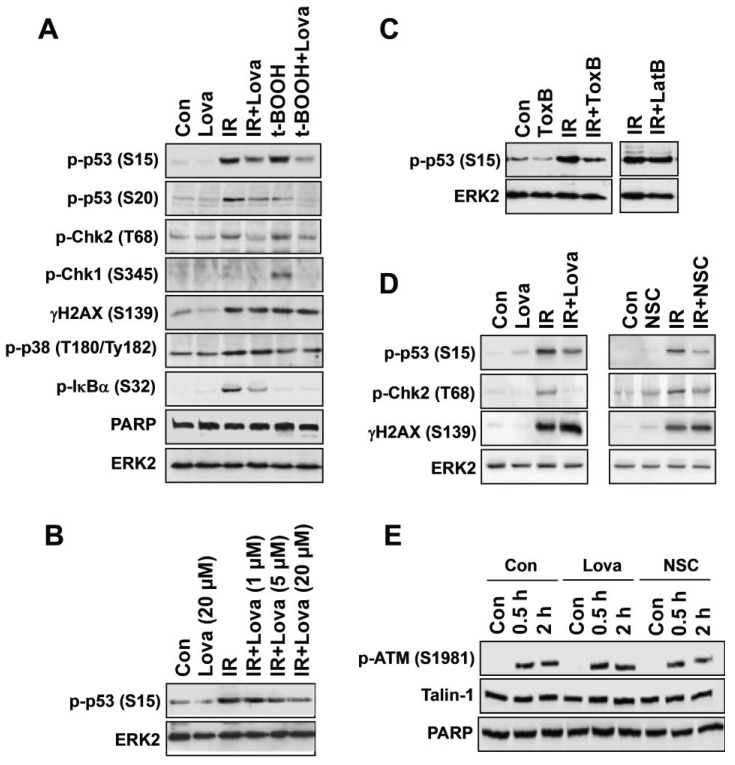
Pleiotropic inhibitory effects of lovastatin on IR-induced mechanisms of the DDR of endothelial-like cells (EA.hy926). (**A**) Logarithmically growing EA.hy926 cells were left untreated or were pre-treated overnight with lovastatin (20 μM) before irradiation (IR) (10 Gy) or treatment with the direct oxidant *tert*-butyl hydroperoxide (t-BOOH) (400 μM). After post-incubation period of 2 h, cells were harvested for Western blot analysis using different phosphospecific antibodies as indicated. PARP and ERK2 expression were analyzed for protein loading control. (**B**) EA.hy926 cells were pre-treated with different concentration of lovastatin (Lova) before irradiation (IR) with 10 Gy. Two hours later, the phosphorylation level of p–p53 was monitored by Western blot analysis. (**C**) EA.hy926 cells were pre-treated for 2 h with the Rho-inhibitory ToxB (10 ng/mL) from *Clostridium difficile* before irradiation was performed (IR, 10 Gy). After further incubation period of 2 h, the level of phosphorylated p53 was determined. For control, cells were also pre-treated with latrunculinB (LatB) (2 μg/mL), which is an inhibitor of actin polymerization. (**D**) To address the question whether Rac1 is a major target for the observed lovastatin effects, the influence of lovastatin and NSC23766 on IR-induced mechanisms of the DDR was investigated in parallel. EA.hy926 cells were either pre-treated overnight with lovastatin (20 μM) or for 2 h with the Rac1-specific small-molecule inhibitor NSC27633 (30 μM) before irradiation was performed (IR, 10 Gy). After further incubation period of 2 h, the level of phosphorylated p53, Chk2 and H2AX was determined. Since lovastatin and NSC23766 revealed quantitatively similar inhibitory effects, we conclude that Rac1 is a major target that mediates the inhibitory effect of the statin on IR-induced DDR. (**E**) EA.hy926 cells were pre-treated overnight with lovastatin (20 μM) or for 2 h with the small-molecule Rac1 inhibitor NSC27633 (30 μM) before irradiation was performed (IR, 10 Gy). After further incubation period of either 0.5 h or 2 h, the level of phosphorylated ATM was determined by Western blot analysis. Expression of talin-1 and PARP protein were analyzed as loading control.

In light of the fact that early Rac1-regulated activation of SAPK/JNK following genotoxic stress can also occur in a DNA damage independent manner [21,38,64], the results of the aforementioned analyses employing Rac1-inhibitory strategies do not provide sufficient evidence that Rac1 affects stress responses originating from damaged DNA. Therefore, the influence of Rho-inhibitory molecules was specifically investigated on mechanisms of the DDR. The results of these studies demonstrated that statins and Rac1-specific small molecule inhibitors (NSC23766, EHT1864) [65,66] also block prototypical signal mechanisms of the DDR, such as the ATM/ATR-regulated phosphorylation of H2AX or p53. For instance, lovastatin largely reduces S139 phosphorylation of H2AX and related formation of γH2AX foci following doxorubicin treatment of human endothelial cells, rat cardiomyocyte cells and human hepatoma cells *in vitro* [55,61,67,68,69] as well as mouse cardiomyocytes and hepatocytes *in vivo* [68,70,71,72,73]. Additionally, a subset of ionizing radiation-induced DDR-related stress responses of endothelial-like EA.hy926 cells, such as the phosphorylation of Chk2 and p53, but not of H2AX, were attenuated by lovastatin if used at a dose of 20 μM (Figure 1A,B). Lovastatin sensitive increase in p-Chk1 level was only found following *tert*-butyl hydroperoxide (t-BOOH) exposure but not IR treatment (Figure 1A). Since the ATM/Chk2 pathway is mainly activated by DSBs and regulates the G1/S checkpoint while activation of ATR/Chk1 reflects replicative stress responses [74], we conclude that t-BOOH is a more potent inducer of replicative stress than IR in endothelial-like EA-hy926 cells. Phosphorylation of IκBα, which is indicative of activation of the transcription factor NF-κB, was specifically stimulated by IR and was sensitive to lovastatin as well. By contrast, IR-stimulated activation of p38 kinase was not affected by the statin (Figure 1A). It is feasible that factors of agent, dose, time of analysis and cell type determine the spectrum of DDR mechanisms that is activated by genotoxins and, accordingly, also influence the inhibitory potency of lovastatin on the DDR. Inhibition of Rac1 by *Cl. difficile* ToxB (Figure 1C) or the small-molecule Rac1 inhibitor NSC23766 (Figure 1D) also attenuated ATM/ATR-regulated phosphorylation of p53 and Chk2, respectively. The inhibitory effect of ToxB is likely independent of changes in actin cytoskeleton because latrunculin B, which inhibits actin polymerization, did not block phosphorylation of p53 (Figure 1C). IR-stimulated phosphorylation of ATM was not affected by the statin (Figure 1E), indicating that it interferes with the DDR downstream of ATM. NSC23766 also failed to block ATM activation (Figure 1E). Lovastatin and NSC23766 also did not affect activation of ATM following treatment of rat cardiomyocytes with doxorubicin, while both inhibitors attenuate the phosphorylation of H2AX in this cell system [67]. Preliminary results from our own ongoing studies indicate that numerous DDR-related stress responses of rat tubular cells following treatment with the anticancer drug cisplatin are also substantially inhibited by lovastatin. Apparently, the inhibitory potency of statins on genotoxin-induced DDR varies in an agent and cell type specific manner. The molecular reason(s) for this startling variation is obscure.

The hypothesis that Rac1 is the most relevant target of the statin-mediated effects on DDR mechanisms is supported by the observation that Cre-mediated deletion of the *rac1* gene in mouse hepatocytes affects the level of hepatocyte DNA damage and the activation of DDR mechanisms following administration of doxorubicin [75] and the chemical carcinogen diethylnitrosamin (DEN) [76]. Statins have also been reported to stimulate Mdm2 phosphorylation, thereby attenuating the p53-regulated response to DNA damage [77]. Apart from influencing mechanisms of the DDR, statins accelerate the repair of oxidative DNA damage in human smooth muscle cells by stabilizing Nijmegen breakage syndrome (NBS)-1 protein, thereby favoring damage recognition by the MRN complex and subsequent activation of ATM [78]. Altogether, these data demonstrate that statin-sensitive, presumably Rac1-regulated, mechanisms do interfere with the DDR machinery upon its stimulation by different types of genotoxins. Yet, since genotoxins also cause pleiotropic effects on membrane and cytosolic structures as repeatedly mentioned before, it can’t be ruled out that signal mechanism regulated by membrane-bound Rac1 are required for an efficient and/or sustained activation of DDR factors. Thus, it is tempting to speculate that a functional cross-talk between nuclear and non-nuclear Rac1-regulated stress signaling mechanisms exists that fine-tunes the DDR. Communication between nuclear and non-nuclear compartments following genotoxic stress is believed to be organized by the non-receptor tyrosine kinase c-Abl, because it shuttles between cytosol and nucleus and gets activated by DNA damage [39]. c-Abl is subject to regulation by ATM in response to ionizing radiation [79] and favors the pro-apoptotic branch of DDR signaling involving p73 [80,81]. Additionally, c-Abl is also required for the formation of Rad51 foci following irradiation [82,83], indicating that it affects homologous recombination repair (HR) [84]. Notably, c-Abl also facilitates activation of ATM/ATR [85], pointing to regulatory feed-back mechanisms. Importantly, the DNA damage-induced nuclear targeting of c-Abl is regulated in a SAPK/JNK/14-3-3 dependent manner [86]. In this context, it should be noted that the stimulating effects of c-Abl on E-cadherin mediated cell-cell adhesions requires Rho GTPase [87] and that the interaction of Bcr-Abl with Vav1, which is a GEF for Rac1, contributes to the activation of Rac1 signaling [88]. Altogether, the data available show that Rac1 interconnects with various key regulators of genotoxic stress responses on different levels.

## 4. Putative Role of Nuclear Rho GTPases in the Regulation of the DNA Damage Response (DDR)

Although these data clearly demonstrate that Rac1 signaling interferes with the regulation of the DDR, they do not provide convincing evidence that actually nuclear functions of Rac1 are involved. A prerequisite for such a primary regulatory role of Rac1 in the DDR is its nuclear localization. Apart from the initially observed cytosolic and membrane localization [12], a nuclear localization of prenylated Rac1 has recently been reported, too [89,90]. In line with this finding, Rac1 harbors a nuclear localization sequence in its C-terminal region and is transported into the nucleus in a karyopherin (KPNA2) dependent manner [89]. Inside the nucleus, Rac1 interacts with numerous nuclear proteins, including IQGAP1-3, RNA helicases, laminB1, histone H3 and topoisomerase type II alpha [89], which is involved in the regulation of DNA replication and transcription. Accumulation of Rac1 in the nucleus preferentially occurs during G2-phase of the cell cycle and promotes cell division [90]. Moreover, IR-induced activation of G2/M checkpoint in MCF-7 cells requires Rac1 protein [91]. Employing CAAX-mutants of Rac1 and using simvastatin to inhibit the prenylation of Rho GTPases, it has been suggested that mainly prenylated Rac1 is present in the nucleus [92].

Recently, it was demonstrated by use of a fluctuation-based method that monomeric and active Rac1 is found in the nucleus following DNA damage, whereas inactive dimeric Rac1 remains in the cytoplasm [93]. The hypothesis of a direct involvement of nuclear Rac1 in the DNA repair and DDR gains substantial support by current data showing that the base excision repair (BER) protein 8-oxoguanin-DNA glycosylase 1 (OGG1) physically interacts with GDP-bound (= inactive) and nucleotide-free Rac1 and, most important, that high intracellular levels of free 8-OxoG base increase the level of GTP-bound (= active) Rac1 [94]. Whether GTP-bound Rac1 is localized at the nuclear membrane or is present in the cytosol under this situation is unclear. It is also uncertain which effector molecule GTP-bound nuclear Rac1 might interact with. Numerous effector molecules have been described for Rac1 [6]. p21-associated protein kinase 1 (PAK1) seems to be of particular relevance, because it has recently been found in the nucleus as well [95] and activation of PAK1/JNK1 by benzo(a)pyrene treatment was reported to trigger apoptosis [96]. Interestingly enough, nuclear translocation of PAK1 can be stimulated by IR and nuclear PAK1 associates with chromatin, thereby causing alterations in gene expression, with the p53 pathway being influenced mostly [95]. Moreover, the RhoA guanine exchange factor Net1 has been identified in the nucleus as well and appears to be a key factor in the cellular response to DNA damage induced by bacterial endonucleases, such as cytolethal distending toxin (CDT) [97]. Interestingly, the activation of RhoA by CDT-induced DNA damage involves the BER factor FEN1 [98], highlighting the functional interference between nuclear Rho-regulated functions and BER-related mechanisms. DNA damage activated RhoA is able to signal to Rho-associated protein kinase (ROCK) [98], which is the key molecule signaling from Rho to the actin cytoskeleton [99]. Importantly, Net1 must leave the nucleus in order to activate RhoA and this relocalization of Net1 requires Rac1 [100]. In this context it should be mentioned that PAK1 inhibits the activity of Net1, again pointing to a complex, so far largely unappreciated, nuclear network of signaling coordinated by Rac1. Taken together these data provide evidence that the Rac1 GTPase interacts with nuclear mechanisms of DNA repair, in particular BER.

Apart from the aforementioned direct interference with BER factors, it is also feasible that Rho GTPases have a more indirect influence on mechanisms of the DDR by regulating chromatin structure and/or nuclear actin cytosekeleton. Thereby, they could control the accessibility of the chromatin for DNA repair and DDR factors or facilitate their intranuclear transport. Bearing in mind that Rho GTPases are key regulators of the actin cytoskeleton [18] and both F-actin and actin-related proteins are present in the nucleus [92,101], this possibility appears realistic. It is feasible that Rho/Rac1-regulated structures of the nuclear cytoskeleton are important for a temporal and spatial distribution of DNA repair and/or DDR factors at the site of damage. This view gains support by recent finding that nuclear Rac1 triggers nuclear shape changes involving actin polymerization [102]. Besides, the actin-binding protein filamin-A interacts with BRCA2 and accelerates the recovery from IR-induced G2/M block [103]. Remarkably, destruction of F-actin structure by cytochalasin D prevents the binding of Ku70 and Ku80 to DSBs [104], supporting the view that polymerized actin participates in the repair of DSBs. Moreover, ATR kinase has recently been shown to relocalize to the nuclear envelope following mechanical stress independent of DNA damage signaling [105]. The latter finding indicates that cytosolic F-actin might act as a sensor of mechanical stress, which is translated into alterations of nuclear plasticity and chromatin remodeling by help of ATR. Last but not least, Rac1 has been shown to be important for the regulation of mitosis [106,107,108]. Interestingly, inactivation of Rac1 seems to be required here [108,109]. Together with the finding that Rac1 enables G2/M checkpoint response [91], it tempting to speculate that Rac1 is particularly relevant for DNA damage-related stress responses of mitotic cells. This hypothesis remains to be scrutinized by appropriate meaningful test systems.

**Figure 2:**
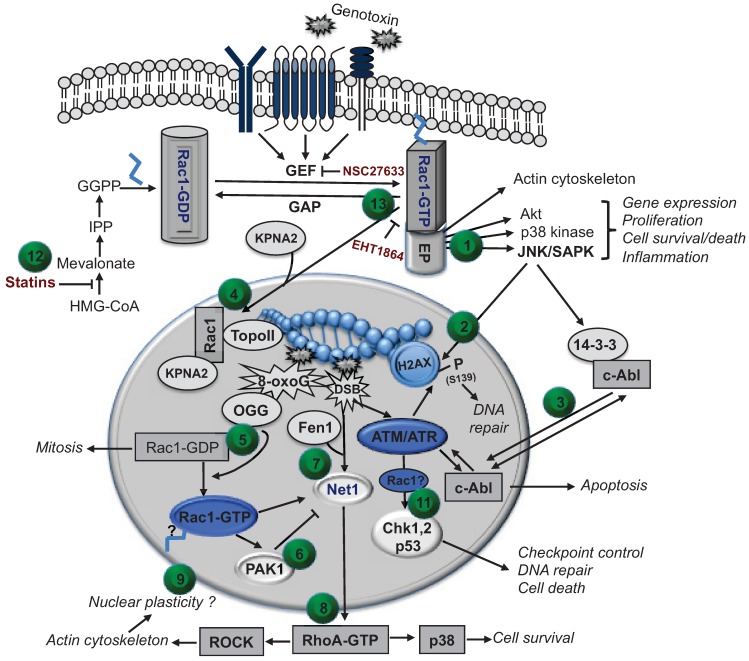
Model of a putative interference of cytosolic and nuclear Rac1 GTPase with mechanisms of the DDR. Exposure to genotoxins results in a rapid activation of membrane-derived stress responses leading to the activation of stress kinases, including Rac1-dependent activation of SAPK/JNK (1), which can phosphorylate H2AX at position S139 as ATM and ATR kinase do (2). The non-receptor tyrosine kinase c-Abl is able to shuttle between cytoplasm and nucleus and is subject to regulation by both SAPK/JNK and ATM (3). c-Abl is believed to trigger DNA damage dependent apoptotic mechanisms via p73. Both prenylated and non-prenylated Rac1 can translocate into the nucleus by help of karyophyrin (KPNA2) (4). Inside the nucleus, Rac1 can be present in GDP- and GTP-bound state and binds to numerous proteins, including topoisomerase II (Topo II) (4). High levels of free 8-oxoG base increase the level of GTP-bound Rac1 (5). Since the Rac1 effector PAK1 is not only involved in signaling to SAPK/JNK but is present in the nucleus as well (6), it is tempting to speculate that PAK1 mediates DDR-related effects of Rac1. Following DNA damage RhoA GEF (e.g., Net1A) get activated (7) leading to a Fen1 dependent activation of the RhoA/ROCK axis (8), which is important for the organization of the actin cytoskeleton (9). Notably, Rac1 controls subcellular localization of Net1 and PAK1 regulates the activity of Net1. Alterations in nuclear actin structure might provide the structural basis for temporal and spacial accessibility of the chromatin to DDR-related factors (10). In addition, there is evidence that Rac1 is required for the activation of checkpoint kinases (Chk1, 2) and p53 downstream of ATM/ATR (11). Taken together, the data available indicate that Rac1-regulated mechanisms coordinate cellular stress responses to genotoxins that originate either from damaged DNA or emerge from the outer membrane. Targeting of Rac1 signaling is therefore suggested as a promising strategy to pharmacologically interfere with the complex stress responses evoked by DNA damaging anticancer drugs and chemical mutagens. In light of the fact that Rac1 can be inhibited by statins (12) as well as by inhibitors of Rac1-specific GEFs (e.g., NSC23766) and GTP-dependent effector coupling (e.g., EHT1864) (13), Rac1 represents a druggable target as well. Therefore, interfering with Rac1-regulated DDR is suggested to be useful in anticancer therapy, by sensitizing tumor cells and/or protecting normal tissue from the adverse effects of conventional (genotoxic) anticancer drugs and radiotherapy. The numbers in the brackets (from 1 to 13) refer to the preceding text passage in the legend and point to the numbered green dots in the figure.

Taken together, the data available to date are strongly indicative of a so far poorly appreciated function of nuclear localized small GTPases, in particular Rac1, in the regulation of cellular responses to genomic DNA damage. Based on published data we suggest that Rac1 is integrated into the complex network of DNA repair and DDR with specific, so far not yet well defined, nuclear functions being involved (Figure 2). Rac1 is suggested as a candidate GTPase that is at the crossroad of a network that coordinates cellular responses to genotoxic stress originating both from damaged DNA and other types of damaged macromolecules.

## 5. Translational Aspects of Rac1 Targeting Strategies in Anticancer Therapy

In view of translational aspects, targeting of Rac1-regulated pro-apoptotic mechanisms of the DDR might be a promising strategy to improve the anticancer efficacy of conventional (genotoxic) anticancer drugs and radiotherapy. Alternatively, therapeutic inhibition of Rac1-dependent pro-apoptotic DDR functions might be a suitable strategy to mitigate normal tissue damage. With respect to drugs that could be clinically used in the middle term, the aforementioned statins are of particular interest for off-label use as Rac1 inhibitory molecules [56], because they are nowadays widely used for lipid lowering purpose already. Apart from increasing the cytotoxic effects of anticancer drugs *in vitro* and *in vivo* [59,110,111], statins are reported to protect normal tissue of the colon from the deleterious effects of radiotherapy [112,113], reduce the nephrotoxicity of cisplatin [114,115] and protect cardiac tissue from the genotoxic and cytotoxic effects of anthracyclines, such as doxorubicin [68,70,73]. Targeting of Rac1 by statins or small molecule inhibitors largely attenuated the formation of DSBs and activation of the DDR following Topo II poisoning by doxorubicin or etoposide [67,69]. Successful attenuation of the clinically most relevant adverse effects of anticancer therapeutics would widen their therapeutic window and, maybe even more important from a patient’s point of view, would bring forward supportive care in cancer by improving quality of life. Moreover, targeting of Rac1 leading to modulated DDR might also be useful for chemopreventive strategies. In this context it should me mentioned that statin use has been associated with a modestly reduce risk of colorectal cancer in recent meta-analysis [116]. Furthermore, statins prevent from chemical colon carcinogenesis *in vivo* [117]. It should also be pointed out that Rac1 is not only a promising target but also a target that might be druggable by different types of drugs. Apart from statins (Figure 2) and prenyltransferase inhibitory molecules (e.g., geranylgeranyltransferase inhibitor (GGTI)), inhibitors of Rac1 GEFs (e.g., NSC23766) and Rac1-effector coupling (e.g., EHT1864) would be compounds of choice for this purpose (Figure 2). Alternatively, GTP-mimetics such as the anti-inflammatory thio-GTP derivative azathioprine, which is already reported to affect Rac1 signaling in CD4+ T-lymphocytes [118], might also be useful. Hence, a plethora of compounds are already available that can target Rac1 signaling. Identifying their usefulness to modulate different branches of the DDR with a clinically beneficial outcome could be subject of forthcoming studies.

## 6. Outlook and Conclusions—Further Validation of Rac1 as Promising Target to Modulate DDR and Repair

Certainly, to scrutinize the impact of Rac1 in the regulation of DDR and DNA repair, more sophisticated experimental test systems are required. One possible option to discriminate between DNA damage dependent and DNA damage independent stress response that Rac1 is interfering with, is the use of specific genotoxins that selectively trigger DNA damage dependent stress responses only. Apart from strategies based on the transfection of restriction enzymes, DNA damaging bacterial toxins such as cytolethal distending toxins (CDT) [119,120,121], are suggested as very powerful and convenient tools to investigate the influence of Rho/Rac1 GTPases on DDR and DNA repair. Importantly, CDT is also particular useful for analyzing mechanisms of the DDR and DNA repair under situation of a sustained induction of low levels of DNA damage [122,123]. Thereby, aspects of thresholds in DDR and repair could be addressed experimentally. Another more laborious but in return meaningful approach is to selectively overexpress or inactivate Rho/Rac1 in the nucleus, for instance by use of expression vectors carrying a nuclear localization sequence (NLS), thereby further favoring nuclear translocation of Rho/Rac1 or Rho-regulatory proteins such as GEFs or Rho-GDI. To our opinion, the ultimate approach to unravel the relevance of nuclear localized Rho/Rac1 GTPase in the regulation of the DDR and DNA repair is a selective expression of wild-type and/or Rho/Rac1 mutants (for instance mutants that lack CAAX box) in the cytosol or nucleus of Rac1 deficient cells, preferentially *rac1−/−* cells. Such cells could be isolated from corresponding *rac1* knock-out animals or could be generated by help of the Crispr/Cas9 technology. Admittedly, such approach might be challenging in light of the fact that conventional genetic knock-out of *rac1* is embryonically lethal [124], making it necessary to employ tissue specific and inducible knock-outs. Moreover, Rac1-deficient cells might be difficult to culture *in vitro* and their phenotype could be misleading since other Rac-isoforms (*i.e.*, Rac2 and Rac3) might functionally compensate for loss of Rac1 and, in general, do cross-react with antibodies considered as Rac1 specific. However, if these pitfalls are carefully addressed, meaningful data regarding the role of nuclear Rac1 in the regulation of mechanisms related to DDR and DNA repair can be generated.
